# Infectious keratitis following photorefractive keratectomy: a 13-year study at a tertiary center

**DOI:** 10.1186/s12348-025-00452-2

**Published:** 2025-01-10

**Authors:** Alireza Attar, Hossein Jamali, Julio Ortega-Usobiaga, Golnoush Mahmoudinezhad, Dagny Zhu, Mohammad Mohammadi

**Affiliations:** 1 Poostchi Ophthalmology Research Center, Department of Ophthalmology, School of Medicine, Shiraz University of Medical Sciences, Shiraz, Iran; 2 Department of Cataract & Refractive Surgery, Clínica Baviera-AIER Eye Hospital Group, Bilbao, Spain; 3 Hamilton Glaucoma Center, Shiley Eye Institute, Viterbi Family Department of Ophthalmology, University of California San Diego, La Jolla, CA USA; 4 NVISION Eye Centers, Rowland Heights, CA USA; 5 School of medicine, Shahid Sadoughi University of Medical sciences, Yazd, Iran

**Keywords:** Infectious keratitis, Refractive surgery, Photorefractive Keratectomy, PRK, Risk factors, Visual acuity, Management, COVID-19

## Abstract

**Introduction:**

Infectious keratitis is a rare but devastating complication following photorefractive keratectomy (PRK) that may lead to visual impairment. This study assessed the clinical features, treatment strategies, and outcomes of post-PRK infectious keratitis.

**Methods:**

This retrospective study was conducted on patients with post-PRK infectious keratitis presenting to Khalili Hospital, Shiraz, Iran, from June 2011 to March 2024. The study was conducted in two stages: the first stage assessed the incidence of post-PRK infectious keratitis among patients who underwent PRK at our center, while the second stage included all patients with post-PRK infectious keratitis, regardless of where their PRK was performed. The following data were collected: demographics, post-surgery presentation time, risk factors, culture results, treatments, follow-up duration, complications, and corrected distance visual acuity (CDVA) at admission and the last follow-up.

**Results:**

Forty-two patients (42 eyes) with a mean age of 28.74 years (male-to-female ratio of 1.2:1) were included. Among 38,938 PRK procedures performed at our center, the incidence of keratitis was estimated to be 0.018% (7/38,938). The odds of keratitis during the COVID-19 pandemic were 7.05 times higher (95% CI: 1.58 to 31.52, *p*-value = 0.015) than outside this timeframe (February 2020 to August 2023). Gram-positive bacteria were the most commonly isolated pathogens in microbiological studies, accounting for 45.2% (19/42) of cases. Early-onset infections were primarily caused by Staphylococcus aureus (9/26, 34.6%), Staphylococcus epidermidis (4/26, 15.4%), and Pseudomonas aeruginosa (4/26, 15.4%), whereas all of the cases with fungi (4/4, 100% (and Acanthamoeba (3/3, 100%) infections caused late-onset infections. All patients received broad-spectrum antibiotic therapy, followed by adjusted treatment based on microbial results. Cases developing endophthalmitis and those not responding to treatment or having non-resolving corneal scars required further interventions, such as penetrating keratoplasty and deep vitrectomy. The mean follow-up duration was 40.81 months, and 97.6% (41/42) of cases experienced CDVA improvement at follow-up.

**Conclusion:**

This long-term study found a post-PRK keratitis rate of 0.018%, with gram-positive bacteria as the most common pathogens. Prompt management and regular follow-up assessments are essential for achieving satisfactory outcomes.

## Introduction

Refractive errors are the leading cause of reversible visual impairment globally [1]. Over the past three decades, the prevalence of refractive errors has significantly increased [2, 3], and it is estimated that by 2050, almost half of the global population will be affected by myopia [2].

Refractive surgery refers to procedures aimed at correcting refractive errors [4]. Since the majority of refractive surgeries are performed for cosmetic purposes, and the candidates are often young individuals in their most productive ages, there is a high expectation for favorable outcomes [5]. Therefore, minimizing complications, optimizing visual outcomes through customized treatments, and improving overall efficacy and safety are essential for individuals undergoing refractive surgeries [4].

Photorefractive keratectomy (PRK), laser-assisted in situ keratomileusis (LASIK), and kerato-refractive lenticle extraction (KLEx) are leading methods of modern corneal refractive surgery, recognized for their high safety and efficacy [4].

PRK is a laser-based refractive surgery that corrects refractive errors by ablating the corneal stroma following epithelium removal [6]. Although performed less frequently than LASIK, PRK may be preferable choice for correcting refractive errors in cases with a predisposition to trauma, thin or irregular corneas, epithelial basement membrane diseases, epithelial abrasions, very flat or very steep corneas, or a higher risk of flap dislocation [6, 7].

Despite significant advancements in safety and precision, modern corneal refractive surgeries are not free from complications [4, 8]. Infectious keratitis is one of the most rare but serious complications after PRK, with the potential to cause significant visual impairment and blindness [9]. Hence, timely diagnosis is crucial for preventing corneal damage and preserving visual acuity (VA) in cases with post-PRK infectious keratitis [9].

Few studies with limited follow-up periods have addressed the incidence rate, clinical features, and outcomes of infectious keratitis following PRK [9–11]. The aim of current study was to evaluate the clinical features, management strategies, and outcomes of infectious keratitis following PRK surgery in one of the largest series, with an extended follow-up period. Our findings may enhance the understanding of this condition and contribute to advancements in clinical practice.

## Methods

The current study was conducted in accordance with the principles of the Declaration of Helsinki, and the study protocol received approval from the Ethics Committee of Shiraz University of Medical Sciences, Shiraz, Iran (ethics code: IR.SUMS.MED.REC.1402.381). Written informed consent was obtained from all participants included in the study.

This retrospective study included individuals with a history of post-PRK infectious keratitis who presented to the ophthalmology departments of Khalili Hospital, Shiraz, Iran, over a 13-year period (from June 27, 2011, to March 17, 2024).

### Study design

The present study was conducted in two stages:

#### Stage 1: Assessing post-PRK infectious keratitis incidence in our center

The first stage aimed to assess the incidence of infectious keratitis after PRK among all patients who consecutively underwent PRK at our hospital during the study period.

#### Stage 2: Evaluating clinical features and outcomes of post-PRK infectious keratitis

The second stage expanded to include all patients diagnosed with infectious keratitis following PRK, regardless of the location where their PRK was carried out.

All patients with a confirmed diagnosis of post-PRK infectious keratitis who were hospitalized, treated at our hospital, and had a minimum of six months of documented post-surgical follow-up results were enrolled in our study. The exclusion criteria included patients diagnosed with non-infectious keratitis and those with less than six months of follow-up. Data were retrieved from the health information system of our center to identify cases who developed infectious keratitis within six months of undergoing PRK surgery.

The following data were reviewed and analyzed: patient gender, age, affected eye, post-surgery presentation time, possible risk factors, the results of microbial cultures, treatments, the last follow-up duration, complications, and corrected distance visual acuity (CDVA) at admission, discharge, and last follow-up visit.

### PRK surgery protocol in our center

Regarding the PRK surgery, preoperatively, the eyes were prepared by cleaning the eyelids with povidone-iodine. The eyelashes were draped, and two drops of 0.5% proparacaine were administered within five minutes prior to the procedure. Additionally, 0.5% topical chloramphenicol was applied to reduce the risk of infection.

During the surgery, the corneal epithelium was removed either by applying 20% alcohol for 20 s or through mechanical debridement using a hockey knife, depending on the surgeon’s technique. Laser ablation was performed on the right eye first, followed by the left, using either the Technolas 217 C or 217-Z-100 excimer laser (Bausch & Lomb). To minimize the chances of developing corneal haze, some surgeons applied topical mitomycin-C (0.02%) directly to the ablated area immediately after the laser treatment.

After surgery, a bandage soft contact lens was placed over the treated eye (for an average of 5–7 days, depending on the completion of corneal re-epithelialization), and patients were prescribed a topical antibiotic and steroid regimen.

This typically included ciprofloxacin 0.3% and betamethasone 0.1%, both used four times daily until the bandage lens was removed. In some cases operated out of our center, depending on the availability of medications in Iran or surgeon preference, alternative antibiotics including chloramphenicol, ofloxacin, or levofloxacin were used in the post-operative treatment regimen. After the contact lens was removed, patients were instructed to follow a tapered course of fluorometholone 0.1% along with preservative-free artificial tears for the next three months.

Moreover, at our center, all PRK patients are routinely followed up at 24 h, three days, seven days, 30 days, 90 days, and 180 days post-surgery to ensure full recovery and monitor for complications.

The CDVA assessments were conducted using the Snellen chart, which was later converted to logMAR values for statistical analysis. VA levels of count fingers (CF), hand motion (HM), light perception (LP), and no light perception (NLP) were given corresponding logMAR values of 1.8, 2.3, 2.3, and 3.0, respectively.

### Diagnosis and management of infectious keratitis

The infectious keratitis diagnosis was established based on a combination of clinical symptoms, slit-lamp examination findings, microbiological results, response to antimicrobial treatment, or a combination of these factors. The clinical diagnostic criteria included corneal infiltrations consistent with infectious keratitis, while excluding non-infectious reasons.

All patients with suspected infectious keratitis underwent corneal cultures, with primary culture panels consisting of Sabouraud agar, Chocolate agar, and Blood agar plates.

Empirical treatment with vancomycin and ceftazidime was initiated until definitive culture results were obtained.

### Data analysis

Descriptive analysis was performed to evaluate the demographic information, predisposing factors, management details, and the incidence of post-PRK infectious keratitis. Fisher’s exact test was employed to assess the significance of the differences in infection rates within and out of the COVID-19 period (February 2020 to August 2023). Furthermore, the Wilcoxon test was used to compare CDVA prior and following treatment. All data were analyzed using SPSS software, version 21.

## Results

### Baseline characteristics

Our study included 42 patients (42 eyes) with the diagnosis of infectious keratitis following PRK. The mean age of the subjects was 28.74 (standard deviation = 5.88, range: 18 to 41) years. The male-to-female ratio was 1.2:1. The patients’ demographics are summarized in Table 1.

**Table 1:** Patients’ demographics

Characteristic	Result
Age (years)	
Mean ± standard deviation Range	28.74 ± 5.95 18–41
**Gender**	
Male Female	23 (54.8%) 19 (45.2%)
**Involved eye**	
Right Left Bilateral	24 (57.14%) 18 (42.86%) 0 (0.00%)
**Surgery Center**	
Our center Referred from other centers	7 (16.67%) 35 (83.33%)
**Time of surgery**	
During COVID-19 era Out of COVID-19 era	12 (28.6%) 30 (71.4%)
**Onset of presentation** ^**a**^	
Early (within 7 days after surgery) Late (from day 8 after surgery)	26 (61.9%) 16 (38.1%)

**a**: The mean time between surgery and presentation was 7.29 days (standard deviation = 2.45, range: 3 to 12 days)

Out of 42 patients with infectious keratitis, PRK had been performed in 7 cases (16.67%) in our hospital, and 35 (83.33%) had been referred from other centers (Table 1).

During the study period, a total of 38,938 PRK procedures (19,469 patients) were performed at our center. Of these, 7 eyes in 7 cases developed infectious keratitis post-surgery. Hence, the overall incidence rate of keratitis at our center was estimated as 0.018% (7/38,938).

During the COVID-19 period (February 2020 to August 2023), the incidence of infectious keratitis (0.13%, 4/3,099) was significantly higher than outside this period (0.02%, 3/16,370) (OR = 7.05, 95% CI: 1.58 to 31.52, *p*-value = 0.015), underscoring the increased risk of keratitis during the pandemic (Table 2).

**Table 2:** Comparison of post-PRK infectious keratitis incidence in our center during and outside the COVID-19 period. (CI: confidence interval)

Time of surgery	Total cases undergoing PRK	Cases with Infectious Keratitis	Incidence rate (%)	Odds ratio	95% CI (lower-upper)	*P*-value (fisher test)
During COVID-19 era	3,099	4	0.13	7.05	1.58–31.52	0.015
Out of COVID-19 era	16,370	3	0.02			

The average time from surgery to presentation was 7.29 days (standard deviation = 2.45, range: 3 to 12). The onset of presentation was early (within one week after PRK) in 26/42 (61.9%) cases and late (more than one week after PRK) in 16/42 (38.1%) (Table 1).

### Risk factors and causative microorganism

Table 3 shows the distribution of the organisms identified among microbial isolates in microbiological studies and the possible risk factors.

**Table 3:** Distribution of risk factors and microorganisms (BCL: bandage contact lens; MRSA: methicillin-resistant Staphylococcus aureus)

Variable		Frequency (%)
Risk factors	Blepharitis	9 (21.43%)
	Dry eye	6 (14.29%)
	Diabetes	4 (9.52%)
	Manipulation of contact lenses	3 (7.14%)
	Ocular trauma	3 (7.14%)
	Washing with water	3 (7.14%)
	Swimming in the sea	2 (4.76%)
	Healthcare-related work	2 (4.76%)
	BCL dislodgment	2 (4.76%)
	Exposure keratopathy due to previous blepharoplasty	1 (2.38%)
	None	7 (16.6%)
Culture results	S. aureus	12 (28.6%)
	S. epidermidis	5 (11.9%)
	Fungal	4 (9.5%)
	Acanthamoeba	3 (7.1%)
	Acanthamoeba + Fungi	1 (2.4%)
	Pseudomonas	4 (9.5%)
	MRSA	2 (4.8%)
	Negative	11 (26.2%)

Cultures were collected from all patients with suspicion of infectious keratitis. Of these, 31 (73.8%) eyes had positive results and 11 (26.2%) cases had keratitis with culture-negative results (Table 3). Of culture-positive eyes, the most common isolated pathogens were Gram-positive bacteria (19 cases, 45.2%), including Staphylococcus aureus (12 cases, 28.6%), Staphylococcus epidermidis (5 cases, 11.9%), and methicillin-resistant Staphylococcus aureus (MRSA) (2 cases, 4.8%) (Table 3).

**Fig. 1:**
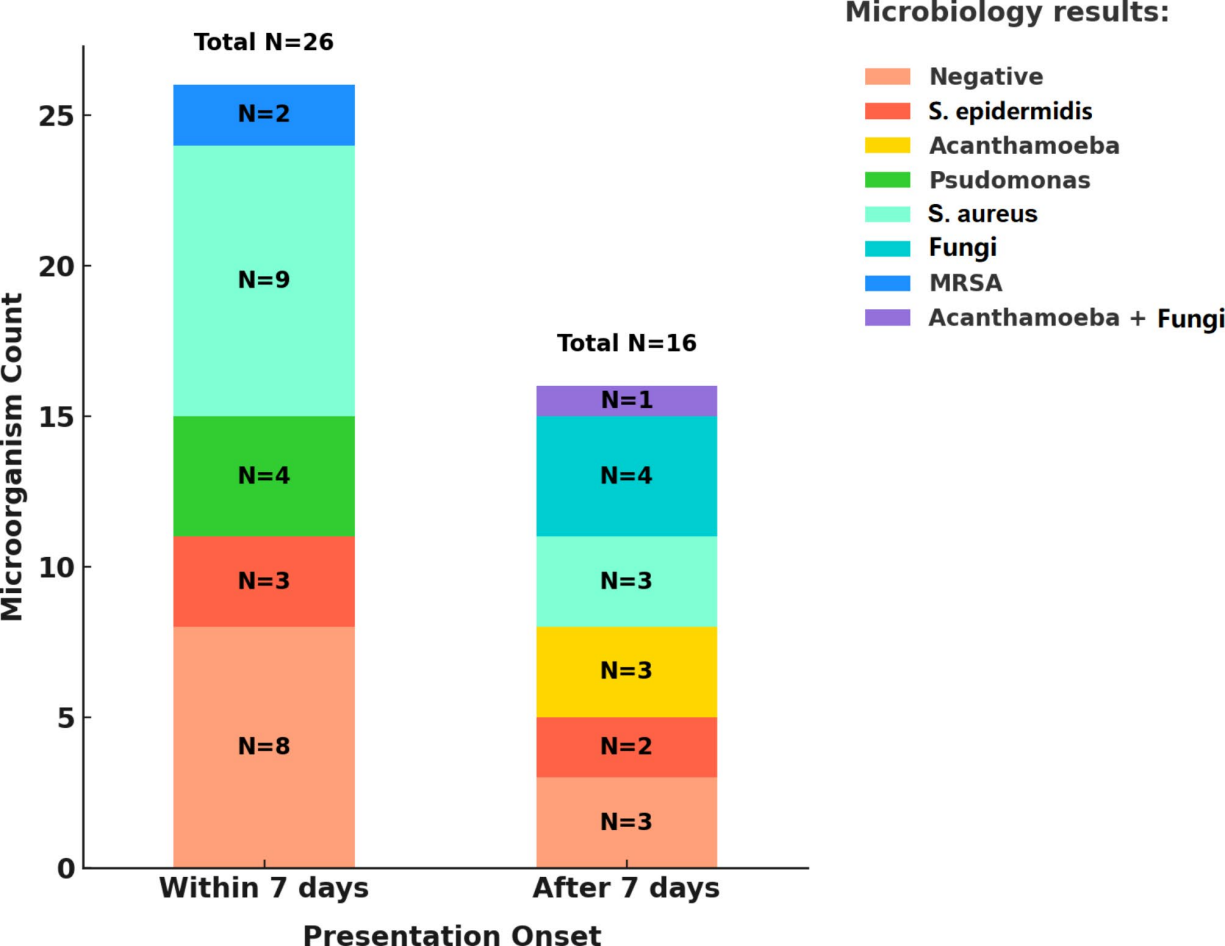
The distribution of organisms identified in microbiological studies according to the presentation onset

Early-onset infections were primarily caused by Staphylococcus aureus, Staphylococcus epidermidis, and Pseudomonas aeruginosa, whereas late-onset infections were more frequently caused by fungal organisms and Acanthamoeba (Fig. 1).

**Fig. 2:**
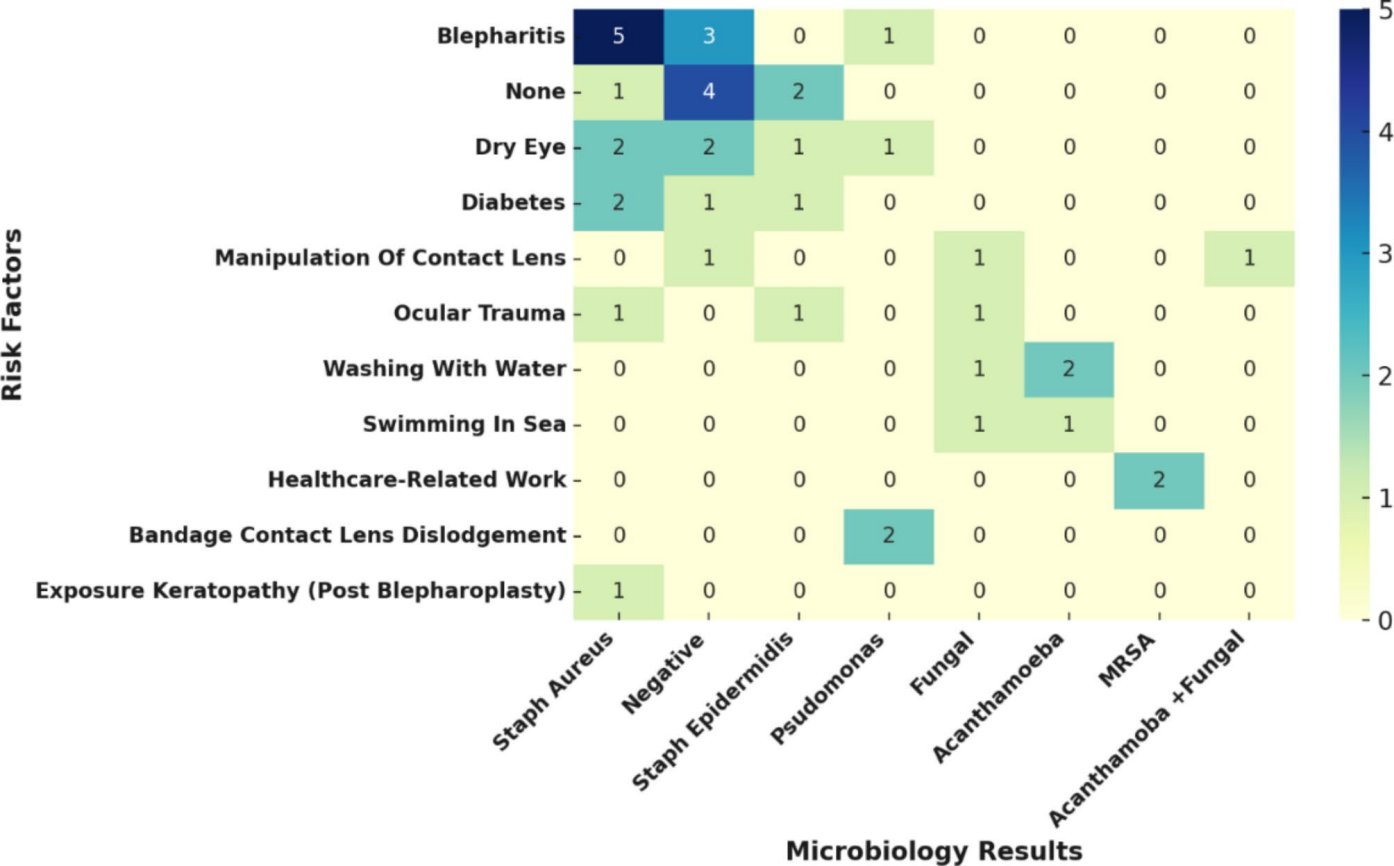
This heatmap illustrates the relationship between and various risk factors. **Note**: The numbers inside the cells represent the counts of cases for each microorganism and risk factor combination

Figure 2 shows the distribution of organisms identified among microbial isolates in microbiological studies across different risk factors.

### Visual acuity

The median CDVA was 1.4 logMAR (IQR 1.477) at admission, 0.698 logMAR (IQR 1.403) at discharge, and 0.349 logMAR (IQR 0.771) at follow-up. CDVA improved significantly from presentation to discharge (*p* < 0.001, Wilcoxon test) and further improved from discharge to last follow-up visit (*p* < 0.001, Wilcoxon test).

The mean (standard deviation) duration of the follow-up period was 40.81 (24.42) months, ranging from 6 to 96 months. Except for one patient with keratitis caused by Acanthamoeba, 41 (97.6%) cases showed improvement in their CDVA after the last follow-up visit. Table 4 shows the CDVA of patients at presentation and follow-up.

**Table 4:** Initial visual acuity versus follow-up corrected distance visual acuity (CDVA). FC: finger counting; HM: hand motion; LP: light perception; NLP: no light perception

Initial VA	Final CDVA					
	≥ 5/10	5/10 ˃ VA ≥ 1/10	FC	HM/LP	NLP	Total
≥ 5/10	1(2.4%)	0(0.0%)	0(0.0%)	0(0.0%)	0(0.0%)	1(2.4%)
5/10 ˃ VA ≥ 1/10	19(45.2%)	1(2.4%)	0(0.0%)	0(0.0%)	0(0.0%)	20(47.6)
FC	1(2.4%)	8(19.0%)	1(2.4%)	0(0.0%)	0(0.0%)	10(23.8)
HM/LP	0(0.0%)	5(11.9%)	6(14.3%)	0(0.0%)	0(0.0%)	11 (26.2)
NLP	0(0.0%)	0(0.0%)	0(0.0%)	0(0.0%)	0(0.0%)	0(0.0%)
Total	21(50.0%)	14(33.3%)	7(17.7%)	0(0.0%)	0(0.0%)	42(100%)

Patients affected by Acanthamoeba, Acanthamoeba + fungi, fungi, Pseudomonas aeruginosa, and MRSA presented with worse presentation CDVA, with a median CDVA of 2.30 (IQR 0.25), 2.30 (IQR 0.00), 2.3 (IQR 0.00), 2.30 (IQR 0.25), and 2.05 logMAR (IQR 0.25), respectively. However, patients with negative cultures and infections with S. aureus and S. epidermidis presented with better CDVA, with medians of 0.698 (IQR 0.47), 1.00 (IQR 0.87), and 1.00 logMAR (IQR 0.30), respectively.

### Management

The mean number of hospitalizations was 14.17 (standard deviation 4.77) days. Table 5 summarizes the frequency of treatment strategies performed for patients.

**Table 5:** Distribution of management strategies performed for post-PRK keratitis (PHMB: Polyhexamethylene biguanide; PK: penetrating keratoplasty). Mean (standard deviation) duration of hospitalization was 14.17 (4.77) days

Treatment		Frequency (%)
Antibiotic therapy	Ceftazidime + Vancomycin + oral Doxycycline	42 (100%)
	Linezolid	2 (4.8%)
	Voriconazole	5 (11.9%)
	Natamycin	5 (11.9%)
Other topical therapy	PHMB	4 (9.5%)
Surgical procedures	PK (at hospitalization)	11 (26.2%)
	Deep vitrectomy (at hospitalization)	2 (4.8%)
	PK (at follow-up)	6 (14.2%)

Empirical antibiotic therapy with fortified ceftazidime (50 mg/ml) and vancomycin (50 mg/ml) eye drops and oral doxycycline (for 2 weeks) were administrated for all the cases.

In five (11.9%) cases where the culture results indicated a fungal nature, an antifungal regimen (voriconazole 1% and natamycin 5%) was started. Polyhexamethylene biguanide (PHMB) (0.02%) was started for 4 (9.5%) cases due to the Acanthamoeba-induced keratitis. In addition, linezolid 0.2% was started for 2 (4.8%) cases of MRSA due to no response to empirical antibiotic therapy.

Two cases (4.8%) developed endophthalmitis following infectious keratitis, necessitating urgent surgical intervention. The cause of keratitis was fungal in one case, while in the other, Pseudomonas aeruginosa was identified as the causative organism. Both patients underwent penetrating keratoplasty (PK) combined with deep vitrectomy in addition to antibiotic therapy.

PK was performed in 11 cases (26.2%). In 9 cases (21.4%), it was performed during the active infection to contain the spread of the infection to the sclera and anterior chamber and to prevent corneal perforation. However, in the other 2 (4.8%) cases, where the patients had endophthalmitis, PK was performed to access better visualization for performing deep vitrectomy.

**Fig. 3:**
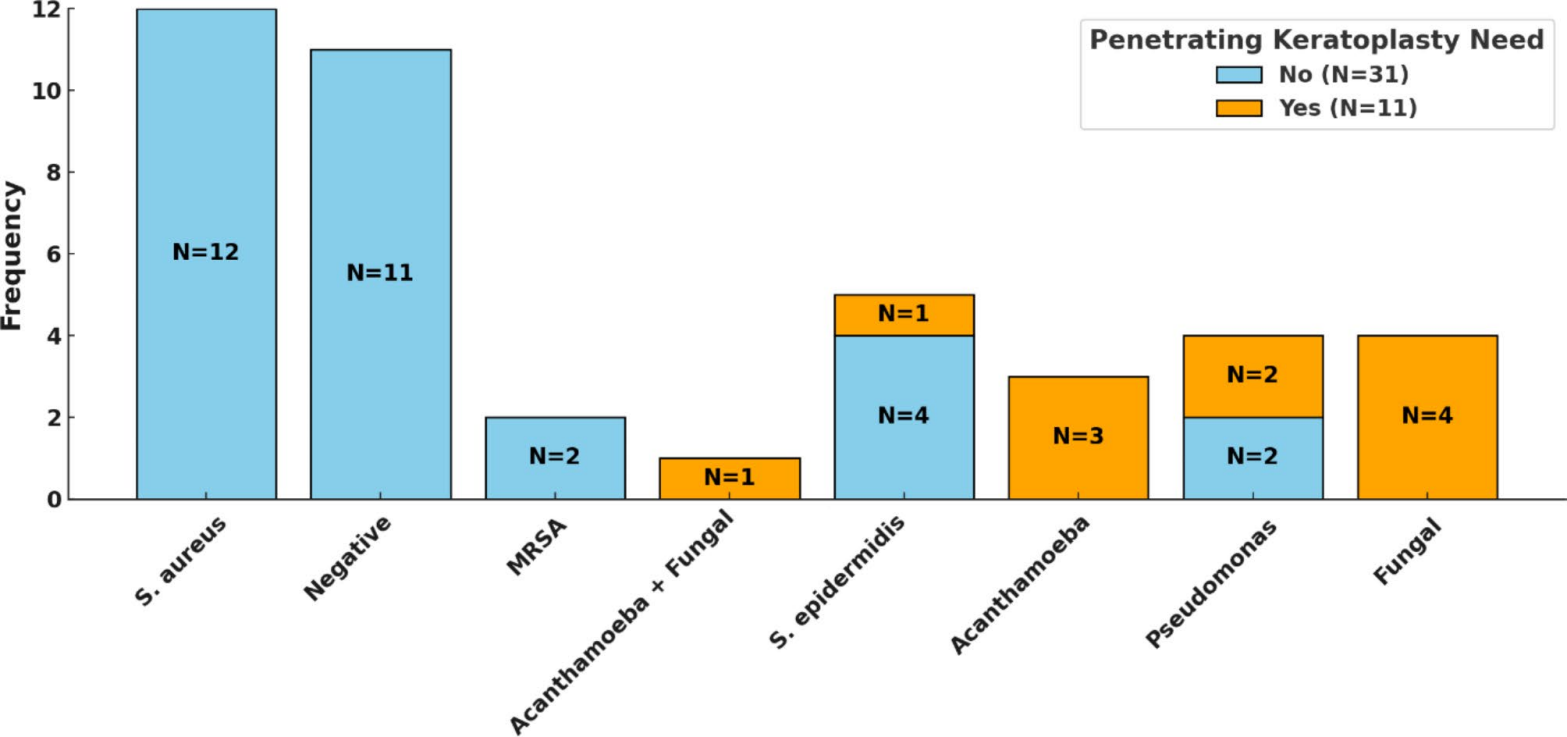
The frequency of different microbiological results according to the need for penetrating keratoplasty during hospitalization

Patients with Pseudomonas aeruginosa, S. epidermidis, Acanthamoeba, and fungal keratitis required PK, whereas all none of the cases with S. aureus, MRSA, and cases with negative-culture needed PK (Fig. 3).

Moreover, in the follow-up visits, PK was performed in 6 cases (14.2%) due to corneal scarring despite the resolution of the infection. The median CDVA for these 6 cases at the last follow-up visit was 0.221 logMAR (IQR 0.147), which was better than the overall group with a median CDVA of 0.349 logMAR (IQR of 0.77).

## Discussion

This retrospective study presented the incidence rate, features, and outcomes of infectious keratitis following PRK in a tertiary hospital over a 13-year period.

In our center, the post-PRK infectious keratitis incidence rate was reported as 0.018%. which was within the range reported in former studies (Table 6) [9–13].

**Table 6:** Incidence of post-PRK infectious keratitis in different studies

Study	Study period	Country	Continent	Number of procedures	Incidence (%)
Leccisotti et al. 2005 [12]	1996–2003	Italy	Europe	10,452	0.02
De Oliveira et al. 2006 [13]	1997–2002	Brazil	South America	4492	0.2
Wroblewski et al. 2006 [10]	1995–2004	USA	North America	25,337	0.02
De Rojas et al. 2011 [15]	2003–2009	Spain	Europe	18,651	0.2
Ortega-Usobiaga et al. 2015 [16]	2010–2013	Spain	Europe	16,674	0.066
Schallhorn et al. 2017 [11]	2008–2015	USA	North America	7434	0.0013
Soleimani et al. 2023 [9]	2014–2020	Iran	Asia	24,986	0.02
Our study 2024	2011–2024	Iran	Asia	38,938	0.018

There is debate concerning the rate of infectious keratitis after photorefractive surgeries when comparing PRK and LASIK [14, 15]. Rojas et al. reported a 5.7-times higher incidence rate of infectious keratitis in patients undergoing surface ablation compared to those undergoing LASIK [15]. The same group [16] reported a decrease in the incidence of post-PRK infectious keratitis from 0.2 to 0.066% when tobramycin and moxifloxacin were used as prophylactic topical antibiotics compared to tobramycin alone; nevertheless, their incidence was 6 times higher than after LASIK (0.011%). This difference has been attributed to factors such as corneal epithelial defects, the use of bandage contact lenses, and corticosteroids used for managing the wound healing process in PRK cases [17, 18]. However, a recent meta-analysis study demonstrated that the incidence rate of keratitis was 4.5 times lower after PRK compared to LASIK [14]. The higher frequency of LASIK procedures and the creation of a corneal flap have been proposed as potential explanations for this observation [14, 19]. Taken together, infectious keratitis remains a significant complication following both PRK and LASIK, highlighting the need for optimizing prevention and management strategies to minimize the risk of adverse outcomes [15, 19].

The presentation time of infectious keratitis following photorefractive surgeries is important in determining its type and may help clinicians make appropriate practical decisions [8]. In our study, 61.9% of cases were classified in the early-onset group. Similarly, it has been reported that approximately 50–60% of infectious keratitis cases present within the first week following surgery, emphasizing the importance of careful monitoring during this period [20–22]. It has been reported that early-onset cases are typically caused by bacteria such as Staphylococcus, Streptococcus, and Pseudomonas, while late-onset cases are often due to slow-growing organisms such as Mycobacteria, Nocardia, Acanthamoeba, and fungi [22, 23]. These findings are consistent with our results.

Regarding the involved eye, Soleimani et al. found that the incidence rate of post-PRK infectious keratitis was higher in the left eye [9]. The time passed since ocular prepping was hypothesized as an explanation for their finding [9]. Similarly, in our study, all cases underwent PRK in the right eye at first. However, the rate of keratitis was higher in the right eye. Therefore, further prospective studies on this matter are recommended.

Patients with post-PRK infectious keratitis typically present with clinical symptoms such as decreased CDVA, eye pain, photophobia, redness, discomfort, discharge, and excess tears [8, 9]. In the initial assessments, corneal infiltrations, epithelial defects, ciliary injections, and hypopyon may be diagnosed [8, 9].

In suspected post-PRK infectious keratitis cases, a culture study of the contact lens and corneal scraping should be performed for accurate diagnosis [24]. The infiltrate in cases of post-PRK infectious keratitis is easily accessible for sampling [20]. Most organisms can be identified through Gram staining and Blood agar cultures. However, in cases with late-onset, it is desirable to also use Ziehl-Neelsen staining, Lowenstein-Jensen media for mycobacteria, and Sabouraud dextrose agar for fungi [25].

Our findings are consistent with previous studies, which identified gram-positive microorganisms as the most common causative agents of post-PRK keratitis [9, 26, 27]. Therefore, prophylactic antibiotics against gram-positive bacteria after PRK have been recommended [9]. No cases of mycobacterial infection were identified in our study. This aligns with previous studies, which have reported that unlike LASIK, surface ablation procedures are rarely related to postoperative atypical mycobacterial infections [9, 24, 28].

Several risk factors for infectious keratitis following PRK, such as contact lens manipulation, dry eye, healthcare professional, blepharitis, ocular trauma, diabetes, frequent hospital encounters, contamination during surgery, and use of corticosteroids have been reported [8]. We identified blepharitis, dry eye, diabetes, swimming in the sea, manipulation of contact lenses, ocular trauma, exposure keratopathy due to previous blepharoplasty, washing with water, healthcare-related work, and bandage contact lens (BCL) dislodgment as possible predisposing factors.

Hence, a thorough ophthalmic examination before the surgery and managing dry eye and blepharitis is essential [24]. The use of the SPEED Questionnaire for ocular surface diseases may help ophthalmologists in identifying these conditions prior to surgery [29].

Studies on contact lens-related keratitis have shown a high prevalence of Pseudomonas aeruginosa induced keratitis [30]. These findings align with our results, which showed that 50% of cases of Pseudomonas aeruginosa infection had a history of contact lens dislodgement. Therefore, regular and frequent corneal examinations are necessary until complete healing of the corneal epithelial defect is achieved, and the bandage contact lens should be removed as promptly as possible [14].

Both MRSA-infected cases in our study were employed in healthcare facilities. This was consistent with previous studies demonstrating an increased risk of MRSA-related infectious keratitis associated with healthcare settings [26, 31].

Soleimani et al. reported that the higher use of face masks during the COVID-19 era was associated with the increased incidence of infectious keratitis following PRK surgery [32]. In their study, the risk ratio and odds ratio for infectious keratitis after PRK were 9.11 and 9.00 during the COVID-19 pandemic, respectively [32]. Similarly, in our center, the incidence of post-PRK infectious keratitis was significantly higher during COVID-19 pandemic, with a sevenfold increased risk compared to outside the pandemic. This may be explained by the redirection of airflow containing oral flora towards the eyes due to mask usage, thereby elevating the infection risk in a cornea that is already compromised [33].

According to previous guidelines, the treatment of infectious keratitis following PRK surgery involves aggressive antibiotic therapy and contact lens removal [34].

It has been suggested that fortified preparations of antibiotics, such as ceftazidime, vancomycin, tobramycin, and cefazolin, are effective against a broad spectrum of gram-positive and gram-negative bacteria [20]. Additionally, the use of topical corticosteroids should be discontinued [35]. In cases of fungal suspicion, the treatment regimen should be adjusted to antifungals such as voriconazole, natamycin, or amphotericin B [22]. Cases with keratitis caused by Acanthamoeba are suggested to be managed with topical chlorhexidine and PHMB [22].

We started fortified ceftazidime and vancomycin as empirical treatment (during hospitalization), followed by oral doxycycline (after discharge) for all the patients. Additionally, we started voriconazole/natamycin and PHMB in cases of keratitis caused by fungal and Acanthamoeba, respectively. Furthermore, linezolid was started for patients with keratitis caused by MRSA. We found that except for one patient (affected by Acanthamoeba), 97.6% of all cases with post-PRK infectious keratitis responded to antibiotic therapy. These findings were in concordance with the results of former studies by Rojas et al. [15], Ortega-Usobiaga et al. [16] and Soleimani et al. [9], who found that nearly all the cases with post-PRK keratitis responded to medical treatment.

Most lesions have been reported to resolve with medical therapy within a few weeks [25]. However, for infections that do not respond to treatment, corneal biopsy or polymerase chain reaction (PCR) may be necessary to identify the causative pathogens [36]. In addition, in severe or non-resolving cases, interventions such as corneal cross-linking or PK may be required [25, 37]. In this regard, patients who developed endophthalmitis (during their hospitalization) and patients with unresolved corneal scarring (at follow-up visits) underwent PK in our study. Notably, we found that the final CDVA of the patients who underwent PK was significantly better than the overall final CDVA of all patients.

Meanwhile, the majority of cases with keratitis caused by fungi, Pseudomonas aeruginosa, Staphylococcus epidermidis, and Acanthamoeba required PK, whereas none of the cases caused by Staphylococcus aureus, MRSA, and negative-culture keratitis required PK.

In our study, 50% of patients achieved a CDVA of 5/10 or better after follow-up. The CDVA of patients at follow-up was worse compared to the results of previous reports, which have reported a CDVA of 20/40 and 20/20 in over 90% and 50% of cases, respectively [9, 10, 15]. This variation may be due to the fact that we only evaluated hospitalized patients and did not include non-hospitalized cases, who naturally have a better initial CDVA and prognosis compared to those requiring hospitalization.

To the best of our knowledge, the present study is the most extensive investigation to date on post-PRK keratitis incidence, with regular and long follow-up periods, that may provide valuable insights for clinicians on managing infectious keratitis. However, the present study has some potential limitations. Firstly, given the retrospective nature of this study, the reported rates of infectious keratitis following PRK surgery may contain inaccuracies, as some affected patients may have sought treatment from other physicians or centers or may not have completed the follow-up period. Secondly, we only included hospitalized cases, excluding those who received outpatient treatment, which may skew the results towards poorer outcomes, and affect the reported incidence of post-PRK corneal infections. Thirdly, our culture studies included some negative results, possibly due to technical issues or prior antibiotic use. Finally, the wide 95% CI for the odds ratio (1.58 to 31.52) comparing the incidence of post-PRK infectious keratitis during and outside the COVID-19 era indicates that the results of this comparison should be interpreted with caution due to the small number of keratitis cases. Therefore, future prospective studies are needed to address these limitations more effectively.

## Conclusions

Infectious keratitis following PRK surgery is a rare but sever complication that may significantly affect CDVA. Our study found a post-PRK keratitis rate of 0.018%, with gram-positive bacteria as the most common pathogen. Prompt management with aggressive broad-spectrum antibiotics, followed by treatment adjustment based on culture results and regular follow-up evaluations, is crucial to achieving satisfactory visual outcomes.

## Data Availability

Requests for materials should be directed to the corresponding authors.

## References

[1] Flitcroft D (2012) The complex interactions of retinal, optical and environmental factors in myopia aetiology. Prog Retin Eye Res 31(6):622–660 22772022 10.1016/j.preteyeres.2012.06.004

[2] Holden BA, Fricke TR, Wilson DA, Jong M, Naidoo KS, Sankaridurg P et al (2016) Global prevalence of myopia and high myopia and temporal trends from 2000 through 2050. Ophthalmology 123(5):1036–1042 26875007 10.1016/j.ophtha.2016.01.006

[3] Vitale S, Sperduto RD, Ferris FL (2009) Increased prevalence of myopia in the United States between 1971–1972 and 1999–2004. Arch Ophthalmol 127(12):1632–1639 20008719 10.1001/archophthalmol.2009.303

[4] Kim T-i, del Barrio Alió, Wilkins JL, Cochener M, Ang B (2019) Refractive surgery. Lancet 393(10185):2085–2098 31106754 10.1016/S0140-6736(18)33209-4

[5] Alexander JK, Davidson RS (2016) Managing expectations in refractive surgery. Int Ophthalmol Clin 56(2):1–17 26938335 10.1097/IIO.0000000000000103

[6] Somani SN, Moshirfar M, Patel BC (2023) Photorefractive Keratectomy. StatPearls Publishing, StatPearls [Internet]

[7] Bower KS, Woreta F (2014) Update on contraindications for laser-assisted in situ keratomileusis and photorefractive keratectomy. Curr Opin Ophthalmol 25(4):251–257 24837576 10.1097/ICU.0000000000000055

[8] Liu J, Guo X, Wei Z, Zhang Y, Zhang Z, Xu X et al (2023) Infectious keratitis after keratorefractive surgery: update and review of the literature. Eye Contact Lens 49(7):275–282 37166228 10.1097/ICL.0000000000000996 PMC10281179

[9] Soleimani M, Keykhaei M, Tabatabaei SA, Shahriari M, Farrokhpour H, Ramezani B et al (2023) Post photorefractive keratectomy (PRK) infectious keratitis; six-year experience of a tertiary eye hospital. Eye 37(4):631–637 35273348 10.1038/s41433-022-02009-2 PMC9998852

[10] Wroblewski KJ, Pasternak JF, Bower KS, Schallhorn SC, Hubickey WJ, Harrison CE et al (2006) Infectious keratitis after photorefractive keratectomy in the United States army and navy. Ophthalmology 113(4):520–525 16488012 10.1016/j.ophtha.2005.09.038

[11] Schallhorn JM, Schallhorn SC, Hettinger K, Hannan S (2017) Infectious keratitis after laser vision correction: incidence and risk factors. J Cataract Refractive Surg 43(4):473–479 10.1016/j.jcrs.2017.01.017 28532931

[12] Leccisotti A, Bartolomei A, Greco G, Manetti C (2005) Incidence of bacterial keratitis after photorefractive keratectomy. SLACK Incorporated Thorofare, NJ, p 96 10.3928/1081-597X-20050101-20 15724694

[13] De Oliveira GC, Solari HP, Ciola FB, Lima ALH, Campos MS (2006) Corneal infiltrates after excimer laser photorefractive keratectomy and LASIK. Slack Incorporated Thorofare, NJ, pp 159–165 10.3928/1081-597X-20060201-14 16523835

[14] Afsharpaiman S, Zare M, Yasemi M, Jamialahmadi T, Sahebkar A (2020) The prevalence of infectious keratitis after keratorefractive surgery: a systematic review and Meta-analysis study. J Ophthalmol 2020(1):6329321 32774907 10.1155/2020/6329321 PMC7407012

[15] de Rojas V, Llovet F, Martínez M, Cobo-Soriano R, Ortega-Usobiaga J, Beltrán J et al (2011) Infectious keratitis in 18 651 laser surface ablation procedures. J Cataract Refractive Surg 37(10):1822–1831 10.1016/j.jcrs.2011.04.037 21865006

[16] Ortega-Usobiaga J, Llovet-Osuna F, Djodeyre MR, Llovet-Rausell A, Beltran J, Baviera J (2015) Incidence of corneal infections after laser in situ keratomileusis and surface ablation when moxifloxacin and tobramycin are used as postoperative treatment. J Cataract Refractive Surg 41(6):1210–1216 10.1016/j.jcrs.2014.09.041 26096523

[17] Cheng KH, Leung SL, Hoekman HW, Beekhuis WH, Mulder PG, Geerards AJ et al (1999) Incidence of contact-lens-associated microbial keratitis and its related morbidity. Lancet 354(9174):181–185 10421298 10.1016/S0140-6736(98)09385-4

[18] Dart J, Radford C, Minassian D, Verma S, Stapleton F (2008) Risk factors for microbial keratitis with contemporary contact lenses: a case-control study. Ophthalmology 115(10):1647–1654e3 18597850 10.1016/j.ophtha.2008.05.003

[19] Santhiago MR, Kara-Junior N, Waring IVGO (2014) Microkeratome versus femtosecond flaps: accuracy and complications. Curr Opin Ophthalmol 25(4):270–274 24837579 10.1097/ICU.0000000000000070

[20] Llovet F, de Rojas V, Interlandi E, Martín C, Cobo-Soriano R, Ortega-Usobiaga J et al (2010) Infectious keratitis in 204 586 LASIK procedures. Ophthalmology 117(2):232–238e4 20006909 10.1016/j.ophtha.2009.07.011

[21] Donnenfeld ED, Kim T, Holland EJ, Azar DT, Palmon RF, Rubenstein JB et al (2005) ASCRS White Paper: management of infectious keratitis following laser in situ keratomileusis. J Cataract Refractive Surg 31(10):2008–2011 10.1016/j.jcrs.2005.10.030 16338575

[22] Das S, Garg P, Mullick R, Annavajjhala S (2020) Keratitis following laser refractive surgery: clinical spectrum, prevention and management. Indian J Ophthalmol 68(12):2813–2818 33229656 10.4103/ijo.IJO_2479_20 PMC7856934

[23] Moshirfar M, Welling JD, Feiz V, Holz H, Clinch TE (2007) Infectious and noninfectious keratitis after laser in situ keratomileusis: occurrence, management, and visual outcomes. J Cataract Refractive Surg 33(3):474–483 10.1016/j.jcrs.2006.11.005 17321399

[24] Soltani Shahgoli S, Cheraqpour K, Soleimani M, Atighehchian M, Tabatabaei SA, Sargolzaeimoghaddam M et al (2023) Post-laser refractive surgery keratitis: a concise narrative review. J Int Med Res 51(10):03000605231206054 37879640 10.1177/03000605231206054 PMC10601402

[25] Haq Z, Farooq AV, Huang AJ (2016) Infections after refractive surgery. Curr Opin Ophthalmol 27(4):367–372 27138638 10.1097/ICU.0000000000000275

[26] Donnenfeld ED, O’Brien TP, Solomon R, Perry HD, Speaker MG, Wittpenn J (2003) Infectious keratitis after photorefractive keratectomy. Ophthalmology 110(4):743–747 12689896 10.1016/S0161-6420(02)01936-X

[27] Leal F, Höfling-Lima AL, Freitas Dd, Campos M (2005) Análise laboratorial das ceratites infecciosas secundárias à Cirurgia refrativa. Arquivos brasileiros de oftalmologia 68:353–356 16059567 10.1590/s0004-27492005000300014

[28] Tabatabaei SA, Soleimani M, Tabatabaei SM, Beheshtnejad AH, Valipour N, Mahmoudi S (2020) The use of in vivo confocal microscopy to track treatment success in fungal keratitis and to differentiate between Fusarium and Aspergillus keratitis. Int Ophthalmol 40:483–491 31701361 10.1007/s10792-019-01209-2

[29] Viriya E, Mah F (2021) Postrefractive infectious keratitis: prevention, diagnosis, management, and prognosis. Curr Opin Ophthalmol 32(4):309–314 33973908 10.1097/ICU.0000000000000775

[30] Musa F, Tailor R, Gao A, Hutley E, Rauz S, Scott R (2010) Contact lens-related microbial keratitis in deployed British military personnel. Br J Ophthalmol 94(8):988–993 20576772 10.1136/bjo.2009.161430

[31] Solomon R, Donnenfeld ED, Perry HD, Rubinfeld RS, Ehrenhaus M, Wittpenn JR Jr et al (2007) Methicillin-resistant Staphylococcus aureus infectious keratitis following refractive surgery. Am J Ophthalmol 143(4):629–634 17320811 10.1016/j.ajo.2006.12.029

[32] Soleimani M, Masoumi A, Farrokhpour H, Keykhaei M, Zeidabadinejad H, Tabatabaei SA (2022) Increased rate of infectious Keratitis after PRK in the COVID-19 era: the possible role of Face masks. J Refract Surg 38(2):78–81 35156460 10.3928/1081597X-20211201-01

[33] Patel SN, Mahmoudzadeh R, Salabati M, Soares RR, Hinkle J, Hsu J et al (2021) Bacterial dispersion Associated with various patient face mask designs during simulated Intravitreal injections. Am J Ophthalmol 223:178–183 33129809 10.1016/j.ajo.2020.10.017 PMC7598764

[34] Silva MVdR, Díez-Feijóo E, Javaloy J, Sánchez-Salorio M (2006) Prophylactic perioperative antiviral therapy for LASIK in patients with inactive herpetic keratitis. SLACK Incorporated Thorofare, NJ, pp 404–406 10.3928/1081-597X-20060401-19 16629075

[35] Stein R (2000) Photorefractive Keratectomy. Int Ophthalmol Clin 40(3):35–56 10941645 10.1097/00004397-200007000-00007

[36] Arnalich-Montiel F, Almendral A, Arnalich F, Valladares B, Lorenzo-Morales J (2012) Mixed Acanthamoeba and multidrug-resistant Achromobacter xyloxidans in late-onset keratitis after laser in situ keratomileusis. J Cataract Refractive Surg 38(10):1853–1856 10.1016/j.jcrs.2012.08.022 22999604

[37] Chang MA, Jain S, Azar DT (2004) Infections following laser in situ keratomileusis: an integration of the published literature. Surv Ophthalmol 49(3):269–280 15110665 10.1016/j.survophthal.2004.02.007

